# An update of the prevalence of osteoporosis, fracture risk factors, and medication use among community-dwelling older adults: results from the Canadian Longitudinal Study on Aging (CLSA)

**DOI:** 10.1007/s11657-022-01073-1

**Published:** 2022-02-04

**Authors:** Caitlin McArthur, Ahreum Lee, Hajar Abu Alrob, Jonathan D. Adachi, Lora Giangregorio, Lauren E. Griffith, Suzanne Morin, Lehana Thabane, George Ioannidis, Justin Lee, William D. Leslie, Alexandra Papaioannou

**Affiliations:** 1 School of Physiotherapy, Dalhousie University, Forrest Building, P.O. Box 15000, Halifax, NS B3H 4R2 Canada; 2 GERAS Centre for Aging Research, Hamilton, Ontario Canada; 3 Health Research Methodology, McMaster University, Hamilton, Ontario Canada; 4 Department of Kinesiology, University of Waterloo, Waterloo, Ontario Canada; 5 Schlegel-UW Research Institute On Aging, Waterloo, Ontario Canada; 6 McMaster Institute for Research On Aging, Hamilton, Ontario Canada; 7 Department of Medicine, McGill University, Montreal, Quebec Canada; 8 Departments of Internal Medicine and Radiology, University of Manitoba, Winnipeg, Manitoba Canada

**Keywords:** Osteoporosis, Fracture risk, Fracture, CLSA

## Abstract

***Summary*:**

The prevalence of self-reported and DXA-confirmed osteoporosis was 7.8% (males 2.2%; females 12.7%), and 3.6% (males 1.2%; females 5.9%), respectively. We found that most community-dwelling older adults at high fracture risk are not taking osteoporosis medication, particularly males. There is a major opportunity for improved primary fracture prevention in the community.

**Purpose:**

To provide an up-to-date prevalence estimate of osteoporosis, fracture risk factors, fracture risk, and the proportion of older Canadians at high fracture risk who are not taking an osteoporosis medication.

**Methods:**

We included Canadian Longitudinal Study on Aging (CLSA) participants: a community-dwelling cohort aged 45 to 85 years who completed the baseline (2015) comprehensive interview and had dual-energy X-ray absorptiometry (DXA) scans (*N* = 30,097). We describe the age- and sex-stratified prevalence of (1) self-reported osteoporosis; (2) DXA-confirmed osteoporosis; (3) fracture risk factors and people who are at high risk (FRAX® major osteoporotic fracture probability ≥ 20%); and (4) people who are at high fracture risk not taking osteoporosis medications. Sampling weights, as defined by the CLSA, were applied.

**Results:**

The mean age of participants was 70.0 (SD 10.3). Overall, 7.8% had self-reported osteoporosis (males 2.2%; females 12.7%) while 3.6% had DXA-confirmed osteoporosis (males 1.2%; females 5.9%), and 2.8% were at high fracture risk (males 0.3%; females 5.1%). Of people who had osteoporosis and were at high risk, 77.3% were not taking an osteoporosis medication (males 92.3%; females 76.8%).

**Conclusions:**

Our study provides an up-to-date prevalence estimate of osteoporosis for community-dwelling older Canadians. We found that most community-dwelling older adults at high fracture risk are not taking an osteoporosis medication, particularly males. There is a major opportunity for improved primary fracture prevention in the community.

**Supplementary Information:**

The online version contains supplementary material available at 10.1007/s11657-022-01073-1.

## Introduction

Osteoporosis is a disease characterized by compromised bone strength and an increased risk for fractures. Osteoporosis-related fractures are associated with significant morbidity, mortality, and economic burden. After a hip fracture, 25% of people require institutionalization [1] and over 30% will die within a year of fracture [2]. In Canada in 2011, the aggregate cost of osteoporosis attributable fractures was $4.6 billion [3]. Resource planning for fracture prevention and treatment relies on accurate prevalence estimates of osteoporosis and fracture risk factors.

Previous studies have estimated the prevalence of osteoporosis in Canada with administrative, clinical, and self-reported data. In 2000, through assessment of bone mineral density (BMD) via dual X-ray absorptiometry (DXA), Tenenhouse et al. [4] estimated that 15.8% of Canadian women and 6.6% of men aged 50 years or older had osteoporosis at the femoral neck or lumbar spine. A population-based study using administrative data found the prevalence of osteoporosis across Canada for those aged 50 years or older to range from 5.6 to 10.5% in 2007/2008 [5] while in 2009 the prevalence of self-reported osteoporosis for adults aged 40 + in the Canadian Community Health Survey was 10.1% [6]. However, these studies were completed 10 to 20 years ago. The aging population is rapidly growing, with nearly one-quarter of the population estimated to be over the age of 65 years by 2030 [7]. Updated prevalence estimates are necessary to ensure resource planning keeps pace with the increasing aging population.

Established fracture risk factors for people with osteoporosis include increasing age, being female, low body mass index, previous fracture, parental hip fracture, smoking, recent systemic glucocorticoid use, rheumatoid arthritis, diabetes mellitus, premature menopause (< 45 years), alcohol use (3 or more units/day), and low femoral neck BMD [8]. Fracture risk can be assessed through tools such as the FRAX® [9], which identifies how likely a person is to experience a fracture in the next 10 years by integrating the effects of multiple risk factors. While the prevalence of diagnosed osteoporosis is important to capture, many fractures occur in the absence of a diagnosis of osteoporosis through BMD [10]. Furthermore, many people who are identified to be at high fracture risk are not offered pharmacological treatment, indicating a missed opportunity for primary fracture prevention [11]. A recent study examining the management of osteoporosis in Canada suggested further areas for research are to understand the proportion of the general population that is at high fracture risk, and that do not receive treatment [12].

Therefore, the objective of the current study is to provide an up-to-date prevalence estimate of osteoporosis (self-reported and DXA-confirmed), fracture risk, fracture risk factors, and the proportion of older Canadians at high fracture risk not taking osteoporosis medications for community-dwelling older adults in the Canadian Longitudinal Study on Aging (CLSA).

## Method

### Data source

The CLSA is a national, stratified, random sample of 51,338 males and females aged 45 to 85 years old across Canada [13]. The tracking cohort of 21,241 was randomly selected from all 10 provinces to participate in telephone interviews only and the comprehensive cohort of 30,097 completed both interviews and physical assessments and biological specimen collection (blood and urine) at one of 11 data collection sites across Canada (Vancouver/Surrey, Victoria, Calgary, Winnipeg, Hamilton, Ottawa, Montreal, Sherbrooke, Halifax, and St. John’s). Three sampling frames were used for recruitment into the CLSA cohort: (1) a subset of participants in Statistic Canada’s Canadian Community Health Survey-Healthy Aging; (2) registries of provincial health care systems; and (3) random digit dialing of landline telephones. In the comprehensive cohort, participants were recruited from individuals living within 25–50 km of a data collection site [14]. Persons excluded from the CLSA were those living in an institution at baseline, full-time members of the Canadian Armed Forces, persons living on federal First Nations reserves and other First Nation settlements, the three northern territories and some remote regions, those unable to respond in English or French, and those with cognitive impairment at baseline. Baseline data were collected between 2011 and 2015. We included all participants who completed the baseline comprehensive interview (face-to-face interview: in-home or data collection site visit) and had DXA data (*N* = 27,685). To ensure generalizability of the sample to the Canadian population, selected weighted demographic and social characteristics of CLSA participants at baseline have been compared with those of the Canadian Community Health Survey Health Aging and Statistics Canada Census 2011 [14]. For most variables, the CLSA participants’ characteristics were generalizable to the Canadian population. However, CLSA cohort participants were more educated, had higher household income, and have a higher percentage of participants who are Canadian-born and rate their general health as very good [14]. Authors hypothesized some of these differences may be because comprehensive cohort participants are committed to a significant amount of time and effort to provide data [14].

### Data analysis

We describe the prevalence of (1) self-reported osteoporosis; (2) DXA-confirmed (femoral neck) osteoporosis according to the World Health Organization definition: osteoporosis (T-score ≤  − 2.5), osteopenia (T-score between − 1.0 and − 2.5), and normal bone density (T-score ≥  − 1.0) as determined by a femoral neck T-score via DXA; (3) each fracture risk factor within FRAX, and people who are at high risk (FRAX major osteoporotic fracture probability ≥ 20%); and (4) people who have osteoporosis and are at high fracture risk who are not taking osteoporosis medications. We did not have access to lumbar spine BMD values and thus could not include them in our definition of osteoporosis. However, the femoral neck has been the most extensively validated site for the definition of osteoporosis and provides a higher gradient of fracture risk than that of many other techniques [15]. Osteoporosis medications were defined from drug identification numbers using the operational structure outlined by the Public Health Agency of Canada (see supplementary file 1) [5]. We did not include over-the-counter supplements like calcium and vitamin D. Fracture risk was determined using the Canadian FRAX risk assessment tool [16]. Participants who have a 20% or greater probability of major osteoporotic fracture were deemed as being at high risk of fracture [16]. We describe the prevalence of each of the following fracture risk factors stratified by sex: age, body mass index, previous fracture, parental hip fracture, current smoking, recent systemic glucocorticoid use, rheumatoid arthritis, diabetes (type 1 insulin-dependent and type 2), premature menopause (< 45 years), alcohol use (3 or more units/day), history of one or more fall within the last 12 months, and femoral neck BMD (g/cm^2^).

Continuous variables were described via mean and standard deviation, while categorical variables were described via count and percent. We calculated a prevalence estimate of osteoporosis diagnosis by self-report and DXA and fracture risk, stratified by age and sex and reported as cases per 1000 persons. We also report the proportion of people who are at high fracture risk and who have osteoporosis confirmed by DXA who are not taking osteoporosis medications. Sampling weights, as defined by the CLSA, were applied [17]. All analyses were conducted in SAS v9.4.

## Results

Table 1 demonstrates that the mean age of our sample was 70.0 (standard deviation 10.3) and 52.5% were female. A higher proportion of females than males self-reported having osteoporosis (males 2.2%, females 12.7%) and had DXA-confirmed osteoporosis (males 1.2%, females 5.9%). Likewise, the prevalence of all identified fracture risk factors was higher for females, except for type 2 diabetes (males 10.4%, females 8.2%), daily alcohol consumption (males 17.2%, females 10.7%), and current smoking (males 46.3%, females 42.0%) where the proportion was higher for men. Using the FRAX score, 2.8% of the entire sample were at high fracture risk (males 0.3%; females 5.1%). The prevalence of osteoporosis and high fracture risk per 1000 persons increased with age for both males and females (Table 2), with the highest prevalence for both sexes among females over the age of 75. Self-reported osteoporosis prevalence was always higher than DXA-confirmed osteoporosis for both sexes across all age groups.

**Table 1:** Demographics of CLSA participants

	All participants	Males	Females
Characteristic	Weighted, *N* (%)	Weighted, *N* (%)	Weighted, *N* (%)
Sex, female	15,788 (52.5)	-	-
Age group			
45–54	11,630 (38.6)	5663 (39.6)	5967 (37.8)
55–64	9501 (31.6)	4545 (31.8)	4956 (31.4)
65–74	5550 (18.4)	2642 (18.5)	2907 (18.4)
75 +	3416 (11.4)	1458 (10.2)	1958 (12.4)
Height (cm), mean (standard error)	168.3 (0.08)	175.5 (0.09)	161.7 (0.07)
Weight, mean (standard error)	80.8 (0.16)	88.6 (0.22)	73.7 (0.19)
BMI, mean (standard error)	28.5 (0.05)	28.7 (0.07)	28.2 (0.07)
Smoking status			
Non-smoker	13,258 (44.1)	5757 (40.2)	7441 (47.1)
Current smoker	13,198 (43.9)	6620 (46.3)	6639 (42.0)
Former smoker	3640 (12.1)	1931 (13.5)	1709 (10.8)
Alcohol consumption			
Never	3809 (13.0)	1631 (11.4)	2177 (13.9)
Less than once a month	4074 (13.9)	1442 (10.1)	2632 (16.7)
1–4 times a month	8427 (28.7)	3823 (26.7)	4604 (29.3)
2–5 times a week	8862 (30.2)	4692 (32.8)	4170 (26.5)
Almost every day	4141 (14.1)	2465 (17.2)	1675 (10.7)
Self-reported osteoporosis	2324 (7.8)	314 (2.2)	2011 (12.7)
Osteoporosis via DXA			
Osteoporosis: DXA T-score < − 2.5	1100 (3.6)	169 (1.2)	932 (5.9)
Osteopenia: DXA T-score − 1.0 to − 2.5	9233 (30.4)	2790 (19.3)	6443 (40.5)
Normal: DXA T-score 1.0 to − 1.0	17,619 (58.0)	10,205 (70.6)	7413 (46.6)
Self-reported lifetime history of fracture	4155 (13.8)	1458 (10.2)	2697 (17.1)
Hip	83 (0.3)	18 (0.1)	64 (0.4)
Arm	194 (0.7)	57 (0.4)	137 (0.9)
Spine	76 (0.3)	33 (0.2)	42 (0.3)
Wrist	824 (2.8)	252 (1.7)	573 (3.8)
Rib	335 (1.1)	165 (1.1)	170 (1.1)
Pelvis	51 (0.2)	16 (0.1)	35 (0.2)
Other	3101 (10.4)	1085 (7.4)	2016 (13.3)
Parental fracture history			
Maternal	2711 (9.0)	1183 (8.3)	1528 (9.7)
Paternal	850 (2.8)	375 (2.6)	475 (3.0)
Recent corticosteroid use	3856 (12.8)	1393 (9.7)	2463 (15.6)
Rheumatoid arthritis	1017 (3.4)	370 (2.5)	647 (4.1)
Premature menopause (< 45 years)*	7901 (50.0)	n/a	7901 (50.0)
Diabetes (type 1)	198 (0.7)	108 (0.7)	90 (0.6)
Diabetes (type 2)	2788 (9.3)	1537 (10.4)	1251 (8.2)
1 or more falls in the last 12 months	1560 (5.2)	629 (4.3)	931 (6.1)
Fracture risk (FRAX score)			
Low	23,588 (77.9)	12,679 (88.1)	10,909 (68.7)
Moderate	3877 (12.8)	623 (4.3)	3253 (20.5)
High	859 (2.8)	41 (0.3)	817 (5.1)
Self-reported osteoporosis medication use	1202 (4.0)	130 (0.9)	1072 (7.1)
Osteoporosis medication use through drug identification number	1070 (3.6)	150 (1.0)	920 (5.8)

^*^% of all females

**Table 2:** Prevalence of osteoporosis and high fracture risk by age and sex per 1000 persons and proportion of people at high risk or with DXA-confirmed osteoporosis who are not taking an osteoporosis medication by age and sex in the CLSA (weighted sample)

	Age and sex									
	All ages		45–54		55–64		65–74		75 +	
Item	Male	Female	Male	Female	Male	Female	Male	Female	Male	Female
			*Prevalence per 1000 persons*							
Self-reported osteoporosis	21.9	127.4	13.1	28.7	20.9	132.4	34.1	218.8	37.7	279.9
DXA T-score < -2.5	11.0	48.8	5.1	21.3	9.9	40.6	14.8	69.8	30.9	122.1
High fracture risk	2.7	39.9	0.0	1.7	1.8	16.5	4.2	54.7	13.0	193.6
			*Proportion of sample*							
DXA-confirmed osteoporosis, no medication	93.5	82.6	100.0	95.9	91.8	86.6	95.1	80.2	90.0	76.1
High fracture risk, no medication	92.7	77.1	-	100.0	88.9	84.5	100.0	81.5	86.4	73.6
DXA-confirmed osteoporosis and high fracture risk, no medication	92.3	76.8	-	100.0	80.0	76.3	100.0	77.1	100.0	76.2

Of people who were at high risk, 97.8% of males and 82.6% of females were not taking an osteoporosis medication. Similarly, of people with DXA-confirmed osteoporosis, 93.5% of males and 82.6% of females were not taking an osteoporosis medication. For people who had both DXA-confirmed osteoporosis and who were high risk, 92.3% of males and 76.8% of females were not taking an osteoporosis medication. These proportions decreased with age for both males and females but were higher for men at all ages and remained above 70% for all ages and both sexes (Table 2).

## Discussion

We provide a new prevalence estimate for individuals at high risk of fracture, and it is lower than previous estimates. However, a major concern is that the vast majority of people who were at high fracture risk were not taking medication for osteoporosis. Moreover, although fewer males were at higher fracture risk than females, a shocking 92% of males at high risk were not taking medication, compared to 77% of females not taking medication.

Our prevalence estimate of DXA-confirmed osteoporosis of 3.6% is lower than previously reported prevalence estimates of osteoporosis in Canada which range from 6.6 to 15.8% [4–6]. There are important differences in data between our study and previous work, namely our study reports DXA-confirmed osteoporosis while previous work relied on self-report [6] or administrative data [5]. Our data suggests that self-reported osteoporosis overestimates osteoporosis confirmed by DXA. Our estimates are also lower than those identified in 2000 by Tenenhouse et al. [4] (femoral neck: males 4.8%; females 7.9%) with data from the Canadian Multicentre Osteoporosis Study (CaMos). The reasons behind our lower estimates are not well understood. However, previous work has demonstrated that BMD has increased and hip fracture rates have decreased over several decades in Canada and the USA for many reasons including a higher prevalence of obesity, more osteoporosis treatment, lower smoking rates, and cohort effects [18]. These factors may be contributing to our observed lower prevalence estimates. Furthermore, the CLSA cohort could be healthier than previously studied populations. As previously described, a higher proportion of the CLSA cohort rate their health as very good compared with the general population and tended to be more educated with a higher income [14]. Thus, our results may reflect a healthy cohort bias.

We identified that for all ages and both sexes, more than 70% of people with DXA-confirmed osteoporosis and who were defined as being at high fracture risk were not taking osteoporosis medication. A similar treatment gap has been observed across 27 European countries, with a mean of 55% of postmenopausal women at moderate, high, and very high fracture risk being untreated with medication [19]. Current osteoporosis guidelines in Canada recommend pharmacologic therapy for people who are at high risk as determined by a validated fracture prediction tool (i.e., FRAX) and for those who have had a low-trauma fracture of the hip or vertebra or more than one low-trauma fracture [11]. There may be valid reasons why a person with osteoporosis who is at high fracture risk may not be taking osteoporosis medications including side effects, financial constraints, lack of efficacy, or inconvenience [20]. However, the fact that the majority of people at high fracture risk are not taking medication despite the clinical practice guidelines indicates a significant missed opportunity for primary fracture prevention. Indeed, previous work has suggested that most osteoporosis quality improvement strategies have focused on secondary prevention, where a person has experienced a recent or prior fracture, with limited attention to individuals without prior fracture [21]. Though the absolute prevalence of osteoporosis (3.6%) may seem low, the high proportion of untreated individuals will cause a significant burden on our healthcare system. Fractures result in substantial pain and disability and cost the healthcare system billions of dollars [3]. Therefore, fracture prevention, whether primary or secondary, should be optimized.

In our study, the prevalence of self-reported osteoporosis was higher for both sexes across all age groups. The measurement error observed in our study is in accordance with previous work that has demonstrated significant disagreement between self-reported and DXA-confirmed osteoporosis [22, 23]. Cadarette et al. [22] found that only 62% with DXA-documented osteoporosis reported their results correctly, with 23% of people with osteoporosis reporting osteopenia and 15% reporting normal bone mass. Likewise, Cunningham et al. [23] demonstrated that many people who self-report osteoporosis are osteopenic indicating confusion between the two conditions [24] or other health conditions like osteoarthritis [25], or poor communication of a clear diagnosis from their health care provider [22]. Factors associated with accurate reporting of a DXA-confirmed osteoporosis diagnosis included female sex, race (non-Hispanic White, Mexican–American, and multiracial), having a lower body mass index, poor health, a history of fracture, and osteoporosis treatment [23]. Individuals who may inaccurately identify a DXA-confirmed diagnosis should be targeted for education about the disease and associated primary or secondary fracture prevention strategies.

An important sex difference was observed in our study: the proportion of males with DXA-confirmed osteoporosis and at high fracture risk and not taking osteoporosis medications was much higher than females across all age groups. Osteoporosis is traditionally underrecognized in males [26], as it is often incorrectly thought to be a woman’s disease. Previous work has demonstrated that the factors associated with osteoporosis care utilization in males were comorbidities, adjuvant hormonal therapy for prostate cancer, vertebral or hip fractures, and glucocorticoid treatment [27]. Thus, males are often not targeted for primary fracture prevention, rather they receive treatment as secondary osteoporosis prevention or when they have secondary osteoporosis. Continued promotion of osteoporosis as affecting both males and females is necessary to decrease the important sex difference highlighted by our study.

A strength of our study is that we used data from the CLSA, which is a population-based national cohort representing seven Canadian provinces. Participants were recruited through stratified random sampling, and we applied the recommended analytical weights to minimize sampling bias. Therefore, our results should be generalizable to the Canadian population between the ages of 45 and 85 years. A limitation of our study is we did not examine factors that could explain why a person would not be taking an osteoporosis medication though they are at high fracture risk. Furthermore, our prevalence estimates rely on femoral neck T-score and do not include the lumbar spine thus likely underestimating total prevalence. Our results are also only generalizable to a fairly healthy community-dwelling older adult population in Canada. The CLSA excluded people at high fracture risk such as those with cognitive impairment and who are institutionalized, and our results cannot be generalized to these groups. Within the baseline CLSA data, we were not able to ascertain which self-reported lifetime fractures were a result of low trauma and at what age they occurred; thus, we were not able to establish how many participants would qualify for a clinical diagnosis based on fracture. However, future work could examine this prevalence with subsequent waves of data collection within the CLSA. Finally, due to the cross-sectional nature of our study, we could not determine how many participants fractured following the assessment. Future work should examine the incidence of fractures for those at high risk and not taking medication.

In conclusion, our study provides an up-to-date prevalence estimate of osteoporosis for community-dwelling older Canadians aged 45 to 85 years. We also demonstrate that most community-dwelling older adults at high fracture risk are not taking osteoporosis medication, particularly males. This presents an opportunity for improved primary fracture prevention in the community.

## Supplementary Information

Below is the link to the electronic supplementary material.

Supplementary file1 (DOCX 74 KB)

## Data Availability

Data are available from the Canadian Longitudinal Study on Aging (www.clsa-elcv.ca) for researchers who meet the criteria for access to de-identified CLSA data.

## Code availability

Analytic code is available upon request.

## Declarations

### Disclaimer

The opinions expressed in this manuscript are the authors’ own and do not reflect the review of the Canadian Longitudinal Study on Aging.
