# Supplementary material for: An update of the prevalence of osteoporosis, fracture risk factors, and medication use among community-dwelling older adults: results from the Canadian Longitudinal Study on Aging (CLSA)
Source: Arch Osteoporos. 2022 Feb 4;17(1):31. doi: 10.1007/s11657-022-01073-1 (PMC8816745; doi:10.1007/s11657-022-01073-1)
Supplement: Supplementary file 1 — Supplementary file1 (DOCX 74 KB) [file 11657_2022_1073_MOESM1_ESM.docx]

| **DIN** | **ACTIVE INGREDIENT** | **PRODUCT NAME** | **STRENGTH** |
| --- | --- | --- | --- |
| 2176017 | ETIDRONATE DISODIUM & CALCIUM CARBONATE | DIDROCAL | 400MG & 500MG |
| 2247323 | ETIDRONATE DISODIUM & CALCIUM CARBONATE | MYLAN-ETI-CAL CAREPAC | 400MG & 500MG |
| 2263866 | ETIDRONATE DISODIUM & CALCIUM CARBONATE | CO ETIDROCAL | 400MG & 500MG |
| 2353210 | ETIDRONATE DISODIUM & CALCIUM | ETIDROCAL | 400MG & 500MG |
| 2324199 | ETIDRONATE DISODIUM & CALCIUM CARBONATE | NOVO-ETIDRONATECAL | 400MG & 500MG |
| 02347989 | ETIDRONATE DISODIUM & CALCIUM CARBONATE | ETIDROCAL | 400MG & 500MG |
| 2233055 | ALENDRONATE SODIUM | FOSAMAX | 5MG |
| 2248251 | ALENDRONATE SODIUM | TEVA-ALENDRONATE | 5MG |
| 2248727 | ALENDRONATE SODIUM | APO-ALENDRONATE | 5MG |
| 2270110 | ALENDRONATE SODIUM | GEN-ALENDRONATE | 5MG |
| 2288079 | ALENDRONATE SODIUM | SANDOZ ALENDRONATE | 5MG |
| 2303035 | ALENDRONATE SODIUM | ALENDRONATE-5 | 5MG |
| 2201011 | ALENDRONATE SODIUM | FOSAMAX | 10MG |
| 2247373 | ALENDRONATE SODIUM | TEVA-ALENDRONATE | 10MG |
| 2248728 | ALENDRONATE SODIUM | APO-ALENDRONATE | 10MG |
| 2270129 | ALENDRONATE SODIUM | MYLAN-ALENDRONATE | 10MG |
| 2288087 | ALENDRONATE SODIUM | SANDOZ ALENDRONATE | 10MG |
| 2303043 | ALENDRONATE SODIUM | ALENDRONATE-10 | 10MG |
| 2245329 | ALENDRONATE SODIUM | FOSAMAX | 70MG |
| 2248730 | ALENDRONATE SODIUM | APO-ALENDRONATE | 70MG |
| 2258110 | ALENDRONATE SODIUM | CO ALENDRONATE | 70MG |
| 2261715 | ALENDRONATE SODIUM | TEVA-ALENDRONATE | 70MG |
| 2270889 | ALENDRONATE SODIUM | RIVA-ALENDRONATE | 70MG |
| 2273179 | ALENDRONATE SODIUM | PMS-ALENDRONATE | 70MG |
| 2275279 | ALENDRONATE SODIUM | RATIO-ALENDRONATE | 70MG |
| 2282763 | ALENDRONATE SODIUM | DOM-ALENDRONATE | 70MG |
| 2282771 | ALENDRONATE SODIUM | PHL-ALENDRONATE | 70MG |
| 2284006 | ALENDRONATE SODIUM | PMS-ALENDRONATE-FC | 70MG |
| 2286335 | ALENDRONATE SODIUM | MYLAN-ALENDRONATE | 70MG |
| 2288109 | ALENDRONATE SODIUM | SANDOZ ALENDRONATE | 70MG |
| 2299712 | ALENDRONATE SODIUM | ALENDRONATE-FC | 70MG |
| 2302004 | ALENDRONATE SODIUM | ALENDRONATE | 70MG |
| 2303078 | ALENDRONATE SODIUM | ALENDRONATE-70 | 70MG |
| 2352966 | ALENDRONIC ACID | ALENDRONATE | 70MG |
| 2248625 | ALENDRONATE SODIUM TRIHYDRATE | FOSAMAX | 70MG/75ML |
| 02401118 | ALENDRONATE SODIUM | ACCEL-ALENDRONATE | 5 MG |
| 02401126 | ALENDRONATE SODIUM | ACCEL-ALENDRONATE | 10 MG |
| 02401134 | ALENDRONATE SODIUM | ACCEL-ALENDRONATE | 70 MG |
| 02381478 | ALENDRONATE SODIUM | ACH-ALENDRONATE | 5 MG |
| 02381486 | ALENDRONATE SODIUM | ACH-ALENDRONATE | 10 MG |
| 02381494 | ALENDRONATE SODIUM | ACH-ALENDRONATE | 70 MG |
| 02258110 | ALENDRONATE SODIUM TRIHYDRATE | ACT ALENDRONATE | 70 MG |
| 02343924 | ALENDRONATE SODIUM TRIHYDRATE | ALENDRONATE | 70 MG |
| 02388545 | ALENDRONATE SODIUM | AURO-ALENDRONATE | 10 MG |
| 02388553 | ALENDRONATE SODIUM | AURO-ALENDRONATE | 70 MG |
| 02308398 | ALENDRONATE SODIUM | DOM-ALENDRONATE-FC | 70 MG |
| 02385015 | ALENDRONATE SODIUM | JAMP-ALENDRONATE | 5 MG |
| 02385023 | ALENDRONATE SODIUM | JAMP-ALENDRONATE | 10 MG |
| 02385031 | ALENDRONATE SODIUM | JAMP-ALENDRONATE | 70 MG |
| 02368552 | ALENDRONATE SODIUM | JAMP-ALENDRONATE | 35 MG |
| 02394855 | ALENDRONATE SODIUM | MINT-ALENDRONATE | 5 MG |
| 02394863 | ALENDRONATE SODIUM | MINT-ALENDRONATE | 10 MG |
| 02394871 | ALENDRONATE SODIUM | MINT-ALENDRONATE | 70 MG |
| 02372304 | ALENDRONATE SODIUM TRIHYDRATE | Q-ALENDRONATE | 70 MG |
| 02384698 | ALENDRONATE SODIUM | RAN-ALENDRONATE | 5 MG |
| 02384701 | ALENDRONATE SODIUM | RAN-ALENDRONATE | 10 MG |
| 02384728 | ALENDRONATE SODIUM | RAN-ALENDRONATE | 70 MG |
| 02270870 | ALENDRONATE SODIUM TRIHYDRATE | RIVA ALENDRONATE | 40 MG |
| 02270889 | ALENDRONATE SODIUM TRIHYDRATE | RIVA ALENDRONATE | 70 MG |
| 02288095 | ALENDRONATE SODIUM | SANDOZ ALENDRONATE | 40 MG |
| 02428717 | ALENDRONATE SODIUM | VAN-ALENDRONATE | 5 MG |
| 02428725 | ALENDRONATE SODIUM | VAN-ALENDRONATE | 10 MG |
| 02428733 | ALENDRONATE SODIUM | VAN-ALENDRONATE | 70 MG |
| 2314940 | ALENDRONATE ACID & VITAMIN D3 | FOSAVANCE | 70MG & 5600 UNIT |
| 2403641 | ALENDRONIC ACID & VITAMIN D3 | TEVA-ALENDRONATE/CHOLECALCIFEROL | 70MG & 5600 UNIT |
| 2276429 | ALENDRONATE SODIUM & VITAMIN D3 | FOSAVANCE | 70MG & 70MCG |
| 2403633 | ALENDRONIC ACID & VITAMIN D3 | TEVA-ALENDRONATE/CHOLECALCIFEROL | 70MG & 2800 UNIT |
| 02405717 | ALENDRONATE ACID & VITAMIN D3 | ALENDRONATE-CHOLECALCIFEROL | 70MG & 2800 UNIT |
| 02405725 | ALENDRONATE ACID & VITAMIN D3 | ALENDRONATE-CHOLECALCIFEROL | 70MG & 5600 UNIT |
| 02429160 | ALENDRONATE ACID & VITAMIN D3 | SANDOZ ALENDRONATE/CHOLECALCIFEROL | 70MG & 5600 UNIT |
| 2242518 | RISEDRONATE SODIUM | ACTONEL | 5MG |
| 2298376 | RISEDRONATE SODIUM | TEVA-RISEDRONATE | 5MG |
| 2298392 | RISEDRONATE SODIUM | TEVA-RISEDRONATE | 35MG |
| 2246896 | RISEDRONATE SODIUM | ACTONEL | 35MG |
| 2302209 | RISEDRONATE SODIUM | PMS RISEDRONATE | 35MG |
| 2319861 | RISEDRONATE SODIUM | RATIO-RISEDRONATE | 35MG |
| 2327295 | RISEDRONATE SODIUM | SANDOZ RISEDRONATE | 35MG |
| 2353687 | RISEDRONATE SODIUM | APO-RISEDRONATE | 35MG |
| 2297787 | RISEDRONATE SODIUM | ACTONEL | 75MG |
| 2316838 | RISEDRONATE SODIUM | ACTONEL | 150MG |
| 2377721 | RISEDRONATE SODIUM | APO-RISEDRONATE | 150MG |
| 2397773 | RISEDRONATE SODIUM | MYLAN-RISEDRONATE | 150MG |
| 2413809 | RISEDRONATE SODIUM | TEVA-RISEDRONATE | 150MG |
| 2239146 | RISEDRONATE SODIUM | ACTONEL | 30MG |
| 2298384 | RISEDRONATE SODIUM | TEVA-RISEDRONATE | 30MG |
| 2370417 | RISEDRONATE SODIUM | ACTONEL DR | 35MG |
| 02406284 | RISEDRONATE SODIUM | AURO-RISEDRONATE | 5 MG |
| 02406292 | RISEDRONATE SODIUM | AURO-RISEDRONATE | 30 MG |
| 02406306 | RISEDRONATE SODIUM | AURO-RISEDRONATE | 35 MG |
| 02442760 | RISEDRONATE SODIUM | AURO-RISEDRONATE | 150 MG |
| 02309831 | RISEDRONATE SODIUM | DOM-RISEDRONATE | 35 MG |
| 02368552 | RISEDRONATE SODIUM | JAMP-RISEDRONATE | 35 MG |
| 02357984 | RISEDRONATE SODIUM | MYLAN-RISEDRONATE | 35 MG |
| 02397773 | RISEDRONATE SODIUM | MYLAN-RISEDRONATE | 150 MG |
| 02358883 | RISEDRONATE SODIUM | NTP-RISEDRONATE | 5 MG |
| 02358891 | RISEDRONATE SODIUM | NTP-RISEDRONATE | 30 MG |
| 02358905 | RISEDRONATE SODIUM | NTP-RISEDRONATE | 35 MG |
| 02427354 | RISEDRONATE SODIUM | PENDO-RISEDRONATE | 35 MG |
| 02309874 | RISEDRONATE SODIUM | PHL-RISEDRONATE | 35 MG |
| 02424177 | RISEDRONATE SODIUM | PMS-RISEDRONATE | 150 MG |
| 02347474 | RISEDRONATE SODIUM | RISEDRONATE | 35 MG |
| 02352141 | RISEDRONATE SODIUM | RISEDRONATE | 35 MG |
| 02370239 | RISEDRONATE SODIUM | RISEDRONATE | 5 MG |
| 02370247 | RISEDRONATE SODIUM | RISEDRONATE | 30 MG |
| 02370255 | RISEDRONATE SODIUM | RISEDRONATE | 35 MG |
| 02411407 | RISEDRONATE SODIUM | RISEDRONATE-35 | 35 MG |
| 02341077 | RISEDRONATE SODIUM | RIVA-RISEDRONATE | 35 MG |
| 2285541 | RISEDRONATE SODIUM & CALCIUM & VITAMIN D3 | ACTONEL SACHET KIT | 35MG & 1000MG /PACK & 880U/ PACK |
| 2279657 | RISEDRONATE SODIUM & CALCIUM | ACTONEL PLUS CALCIUM | 35MG & 500MG |
| 02362414 | RISEDRONATE SODIUM & CALCIUM | PMS-RISEDRONATE PLUS CALCIUM | 35MG & 500MG |
| 2247585 | CALCITONIN | APO-CALCITONIN NASAL SPRAY | 200U |
| 2261766 | CALCITONIN | SANDOZ CALCITONIN NS | 200U/SPRAY |
| 2311046 | CALCITONIN | PRO-CALCITONIN - 200 | 200U/SPR |
| 2240775 | CALCITONIN | MIACALCIN NASAL SPRAY 200IU | 200UNIT/ACT |
| 2239028 | RALOXIFENE HYDROCHLORIDE | EVISTA | 60MG |
| 2358921 | RALOXIFENE HYDROCHLORIDE | PMS-RALOXIFENE | 60MG |
| 2279215 | RALOXIFENE HYDROCHLORIDE | APO-RALOXIFENE | 60MG |
| 2312298 | RALOXIFENE HYDROCHLORIDE | TEVA-RALOXIFENE | 60MG |
| 02358840 | RALOXIFENE HYDROCHLORIDE | ACT RALOXIFENE | 60MG |
| 02415852 | RALOXIFENE HYDROCHLORIDE | RALOXIFENE | 60MG |
| 2254689 | TERIPARATIDE | FORTEO | 250MCG/ML |
| 2269198 | ZOLEDRONIC ACID | ACLASTA | 5MG/100ML |
| 2408082 | ZOLEDRONIC ACID | ZOLEDRONIC ACID INJECTION | 5MG/100ML |
| 2415100 | ZOLEDRONIC ACID | TARO-ZOLEDRONIC ACID | 5MG/100ML |
| 02408449 | ZOLEDRONIC ACID | ACT ZOLEDRONIC ACID | 5 MG / 100 ML |
| 02421720 | ZOLEDRONIC ACID | ZOLEDRONIC ACID - A | 5 MG / 100 ML |
| 02422433 | ZOLEDRONIC ACID | ZOLEDRONIC ACID INJECTION | 5 MG / 100 ML |
| 2343541 | DENOSUMBA | PROLIA | 60MG/ML |
| 02343568 | DENOSUMBA | PROLIA | 60MG/ML |
